# IMPLANT: a new technique for transgene copy number estimation in plants using a single end-point PCR reaction

**DOI:** 10.1186/s13007-022-00965-0

**Published:** 2022-12-09

**Authors:** Jonas De Saeger, Jihae Park, Kai Thoris, Charlotte De Bruyn, Hoo Sun Chung, Dirk Inzé, Stephen Depuydt

**Affiliations:** 1 Laboratory of Plant Growth Analysis, Ghent University Global Campus, Incheon, 406-840 South Korea; 2 Department of Plant Biotechnology and Bioinformatics, Ghent University, 9052 Ghent, Belgium; 3 Center for Plant Systems Biology, VIB, 9052 Ghent, Belgium; 4 Department of Marine Sciences, Incheon National University, Incheon, 406-840 South Korea; 5 Present Address: Laboratory of Molecular Biology, Wageningen University & Research, 6708 PB Wageningen, The Netherlands

**Keywords:** Copy number determination, Competitive PCR, T-DNA insertions, IMPLANT, Genetically modified plants

## Abstract

**Background:**

Copy number determination is one of the first steps in the characterization of transgenic plant lines. The classical approach to this, Southern blotting, is time-consuming, expensive and requires massive amounts of high-quality genomic DNA. Other PCR-based techniques are either inaccurate, laborious, or expensive.

**Results:**

Here, we propose a new technique, IMPLANT (**I**nsertion of co**m**petitive **P**CR ca**l**ibr**a**tor for copy **n**umber es**t**imation), a competitive PCR-based technique in which the competitor (based on an endogenous gene) is also incorporated in the T-DNA, which then gets integrated in the genome together with the gene of interest. As the number of integrated competitor molecules directly corresponds to the number of transgene copies, the transgene copy number can be determined by a single PCR reaction. We demonstrate that the results of this technique closely correspond with those obtained by segregation analysis in Arabidopsis and digital PCR In rice, indicating that it is a powerful alternative for other techniques for copy number determination.

**Conclusions:**

We show that this technique is not only reliable, but is also faster, easier, and cheaper as compared with other techniques. Accurate results are obtained in both Arabidopsis and rice, but this technique can be easily extended to other organisms and as such can be widely adopted in the field of biotechnology.

**Supplementary Information:**

The online version contains supplementary material available at 10.1186/s13007-022-00965-0.

## Background

Transgene copy number is an important characteristic of transgenic plant lines. In most applications, the integration of a single copy is the most desired outcome, as the presence of multiple copies is more likely to lead to gene silencing [1]. In subsequent generations, the zygosity of these transgenes can also influence the phenotype: homozygotes have different characteristics as compared with heterozygotes. Although there have been only few reports about a gene dosage phenomenon in transgenic plants [2, 3], it can be an important factor, depending on the inserted transgene.

Several methods have been developed to determine the copy number and/or zygosity in plants. Traditionally, Southern blotting has been used for this purpose, but this technique is time consuming, expensive, and not very sensitive. It also needs large amounts of DNA: typically several tens of micrograms. While several detection methods exist nowadays, radioactive probes—which offer high signal to noise ratios but are hazardous to work with—are still commonly used [4]. Moreover, when the goal is to determine zygosity, band intensities of parental generations and their offspring need to be compared directly on the same blot, as the pattern is identical [5, 6]. Several other methods have been proposed ranging from phenotypic methods to next-generation-sequencing [7]. For a recent overview of methods that can be used, we refer to the review of Passricha and coworkers [3].

Some of the earliest polymerase chain reaction (PCR)-based methods that have been used to determine copy numbers are thermal asymmetric interlaced (TAIL)-PCR, inverse PCR, ligation-PCR and variations thereof [8–10]. Next to an estimate of the copy number, these methods also allow to characterize the genomic sequences of the flanking regions. The downside is that these methods are laborious, and even when multiple different primers/enzymes are used, detection of all DNA integrations is not guaranteed.

Quantitative PCR (qPCR) seems like an ideal technique to quantify the number of DNA copies, but due to random PCR variation, it is difficult to accurately measure the difference between one and two transgene copies. As such, it is only useful for an initial screening, i.e., to identify plants that contain a low number of transgene copies [11, 12]. The use of statistical models may help to gain confidence in the estimate, but many parameters must be tightly controlled to get reliable results [13]. An interesting variation however is Standard Addition qPCR (SAQPCR), in which a known amount of a DNA standard is added to the PCR reaction [14]. This method has not gained much traction partly because it also necessitates accurate dilutions of genomic DNA and a DNA standard, and because it relies on complex equations to estimate the copy number, making it a rather cumbersome technique. Droplet digital PCR (ddPCR) is a relatively recent technique in which the PCR reaction is partitioned in minuscule droplets, after which fluorescence is measured in each droplet independently [15]. Although it was originally intended for (c)DNA quantification, several studies indicate that ddPCR is just as reliable for copy number estimation as Southern blotting, but much faster [9, 16]. Despite the good accuracy, ddPCR is sensitive to template DNA quality, and is also at least one order of magnitude more expensive than conventional PCR reactions [17, 18].

Perhaps surprisingly, end-point PCR is more accurate than qPCR to determine copy numbers, at least when it is used in the form of competitive PCR (cPCR). In this method, a competitor is created that shares primer-binding sites with the fragment of interest (or target), and that is amplified with a similar efficiency [19]. This similar efficiency necessitates that both amplicons are similar in terms of length and GC-content. This competitor is added to the PCR reaction together with the genomic DNA containing the target sequence. Because the competitor and the target are amplified in the same reaction, they are subject to the same conditions and amplify at a similar rate. Several formats have been used to determine the copy number, by doing two consecutive cPCRs to first accurately determine the gDNA amount and then the transgene copy number [20], by using multiple cPCRs with different competitor concentrations, or by performing two cPCRs: one with a high and one with a low amount of competitor to generate a standard curve based on mathematical modelling [21]. These methods still require a lot of handling and manipulation to come to a copy number estimate. Another end-point PCR method is conceptually similar to cPCR but uses two different primer sets, one for a reference gene and one for the target, with similar melting temperatures [22]. While this method is promising, it does require a careful selection of amplicons to be amplified and a concomitantly precise primer design. Moreover, PCR efficiency with these different primer sets will also depend on the buffer and on the PCR amplification bias of the DNA polymerase that is used [23, 24].

In the light of the shortcomings of the existing methods, we introduce IMPLANT (**I**nsertion of co**m**petitive **P**CR ca**l**ibr**a**tor for copy **n**umber es**t**imation), a new technique that uses a single end-point cPCR reaction to determine copy numbers. This method is based on cPCR, which has proven to be a reliable method and importantly, takes away cumbersome quantification of DNA and multiple PCR reactions. Our proposed method relies on the introduction of a construct containing both the gene of interest and a competitor based on an endogenous sequence. After integration of this construct into the plant genome, the amplicon amount of the competitor can be directly compared with that of the endogenous sequence that will be present in two copies in diploid organisms. After PCR, the concentration of the two amplicons can be analyzed with capillary gel electrophoresis and the ratio between the peaks will reflect the copy number of the plant. The method was benchmarked against ddPCR and was shown to give accurate results.

## Results

### Principle of the IMPLANT procedure

**Fig. 1:**
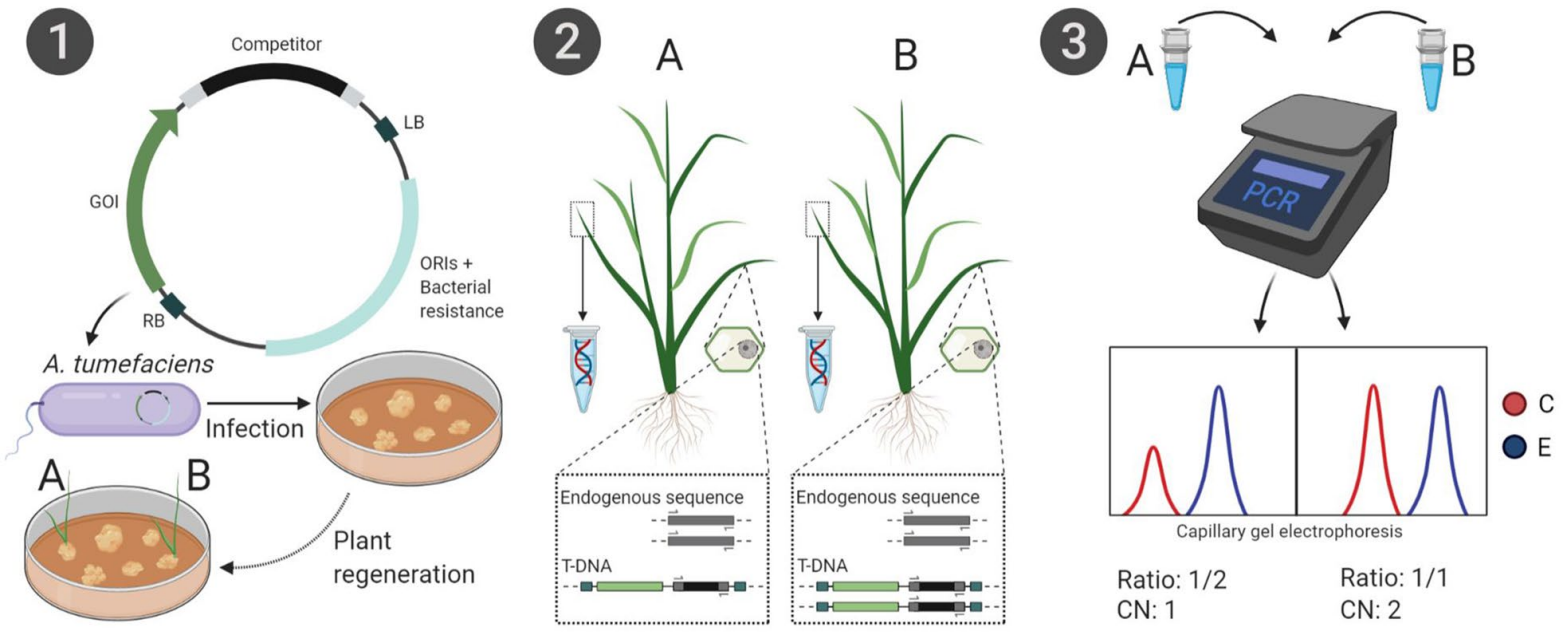
The IMPLANT protocol. In step 1, transgenic plants are generated that contain the gene of interest (GOI) and a selection marker gene with a built-in competitor sequence. This internal sequence can be any sequence, as long it has a similar GC content and can be distinguished from the endogenous gene after amplification with the same primers (indicated in gray) by amplicon size or by size differences after restriction enzyme digestion. For illustrational purposes, we depicted *Agrobacterium*-mediated transformation with a T-DNA binary vector in a callus-based system with plant regeneration after selection with the marker. In step 2, two different transgenic plants are shown. One plant harbors a single transgene copy (**A**), while the other contains two transgenes (**B**). The primer pair is indicated on both the endogenous sequence and the competitor present on the transgene. DNA is harvested from both plants to use in the PCR reaction in step 3. Because the endogenous gene and the transgene will compete for the same primers, the ratio between the peak heights (after capillary electrophoresis) will be proportional to the copy number

We designed IMPLANT as an efficient and straightforward end-point PCR system to determine transgene copy numbers (Fig. 1). It is based on a cPCR reaction, but with the integration of both the gene of interest and the competitor (based on and competing with an endogenous gene for amplification) into the plant genome. Because similar amplification efficiencies using only one primer pair for the competitor and endogene are required, the GC content and length of both amplicons have to be similar when designing the competitor sequence. Therefore, a size difference of maximally 10% is required to discriminate between both amplicons when using gel electrophoresis for amplicon quantification. Because both DNA species are integrated into the plant genome, DNA standards containing the competitor are not necessary, and due to the competitive nature of the reaction, end-point PCR can be used without any danger of PCR cycle-artifacts. For the calculation of the copy number, the signal intensity of the competitor is set relative to the intensity of the endogenous sequence. Because of differences in amplification efficiency between the endogenous sequence and the competitor, this value needs to be multiplied with an empirically derived correction factor. The resulting value is then rounded up or down to the nearest integer to obtain the final copy number estimate (see Materials and methods). When setting up the assay for a given plant species, competitor, and PCR conditions, it is advisable to confirm the results with another technique such as ddPCR or Southern blot analysis. After the assay is set up, the same correction factor can be used for subsequent experiments.

IMPLANT is proposed here in combination with *Agrobacterium*-mediated transformation, and tested in both Arabidopsis and rice, but it is equally applicable to other transformation methods that give rise to stably transformed plants, or even to other organisms. For the Arabidopsis experiments, we used a quick-and-dirty DNA extraction protocol [25], but for most plant species this is unfortunately not possible. For the rice experiments, we therefore used spin column-purified DNA as the PCR template, although some relatively quick methods such as the Edwards’ protocol [26] might also give reasonably good results. For high-throughput screening of single T-DNA copy plants, even these quick methods are not only labor intensive, but they also require several pipetting steps that increase the chance of sample mix-ups. Therefore, we also used IMPLANT in a direct PCR format, which will increase the ease of use.

### IMPLANT can accurately determine transgene copy numbers in Arabidopsis

**Fig. 2:**
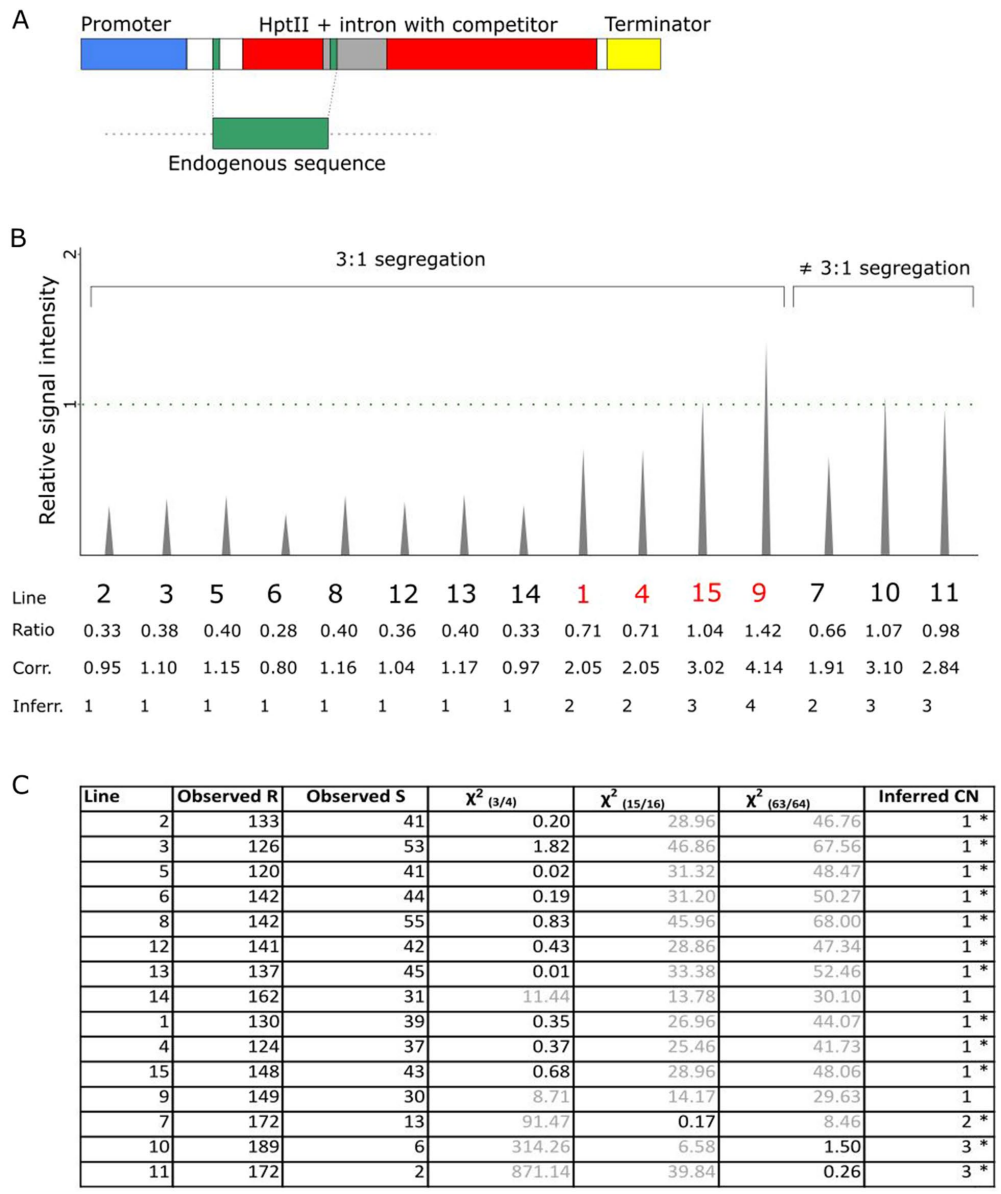
IMPLANT results and segregation analysis in *A. thaliana*. **A**. Overview of the modified hygromycin resistance marker in pICSL11059, with indication of the competitor (408 bp) and the amplified fragment of the endogenous *Arabidopsis* gene *AT2G28000* (370 bp). **B**. IMPLANT results using capillary gel electrophoresis. The signal intensity of the competitor is set relative to the intensity of the endogenous sequence (dotted green line), named “Ratio”. The correction factor was empirically determined and set to 2.9 and the corrected ratios (“Corr.”) were rounded up or down to the nearest integer to obtain the inferred copy number (“Inferr.”). The segregating T2 lines that follow a 3:1 segregation ratio in our segregation analysis, but that show the presence of more than one T-DNA copy, are indicated in red. **C**. Chi square calculation of segregation analysis results. The chi-squared value is calculated for three cases, when there is one transgene-containing locus, leading to three out of four resistant plants, as well for two and three loci. When the calculated value was significant it is printed in black, if not, in gray. The significance of the copy number estimation is denoted by an asterisk (at the 5% significance level). R, resistant; S; sensitive; χ2, Chi square; CN, copy number

To provide proof of concept for IMPLANT to accurately determine copy numbers, we first tested it in transgenic *Arabidopsis thaliana* lines. An endogenous amplicon of 370 bp (GC: 47.3%) was selected within the *SCHLEPPERLESS* (*AT2G28000*) gene (Additional file 1: Figure S1A) and the same primer-binding sites were cloned inside pICSL11059 [27], a binary vector that contains a hygromycin plant resistance marker. The forward primer was cloned upstream of the start codon and the reverse primer inside the catalase intron of the hygromycin resistance gene (Fig. 2A, Additional file 1: Figure S1B), ensuring functionality for hygromycin selection. As such, a competitor amplicon of 408 bp with a similar GC content (51.5%) was created, which can be distinguished from the endogenous 370-bp *SCHLEPPERLESS* amplicon by size after amplification with the same primers. Note that our modified vector pICSL11059_AtIMPLANT, only differs by 43 bp with the parental vector. After transformation into Arabidopsis, we used IMPLANT for copy number estimation (Fig. 2B).

A segregation analysis was also done on the same lines. As can be seen in Fig. 2C, most of the generated lines contain one single T-DNA locus according to segregation analysis. This could also be concluded from the IMPLANT protocol, which generated results that deviated at most 0.2 (all values between 0.8 and 1.17) from the expected ratio of 1 for these lines. Note that some of the lines that segregated in a 3:1 ratio however, contained multiple T-DNA copies (most likely tandem insertions that cannot be picked up by segregation analyses alone) (line #1, #4, #15 and #9). IMPLANT is able to discriminate between such lines and single inserts, pointing to a distinct advantage of using IMPLANT as opposed to segregation analysis.

The three lines in this study that segregated in a ratio different from 3:1 (#7, #10, #11), were also identified by our IMPLANT protocol, and contained either two or three copies of the transgene.

To further validate our IMPLANT protocol, we performed a follow-up experiment with the T1 progeny of independent T0 lines. We performed copy number analysis with IMPLANT, and instead of segregation analysis, we made use of ddPCR to compare the obtained copy number estimates. In this experiment, we again found that IMPLANT could reliably distinguish between single and double transgene containing plants, as benchmarked with ddPCR (Additional file 1: Figure S4).

### IMPLANT also works in the monocot species *Oryza sativa*

**Fig. 3:**
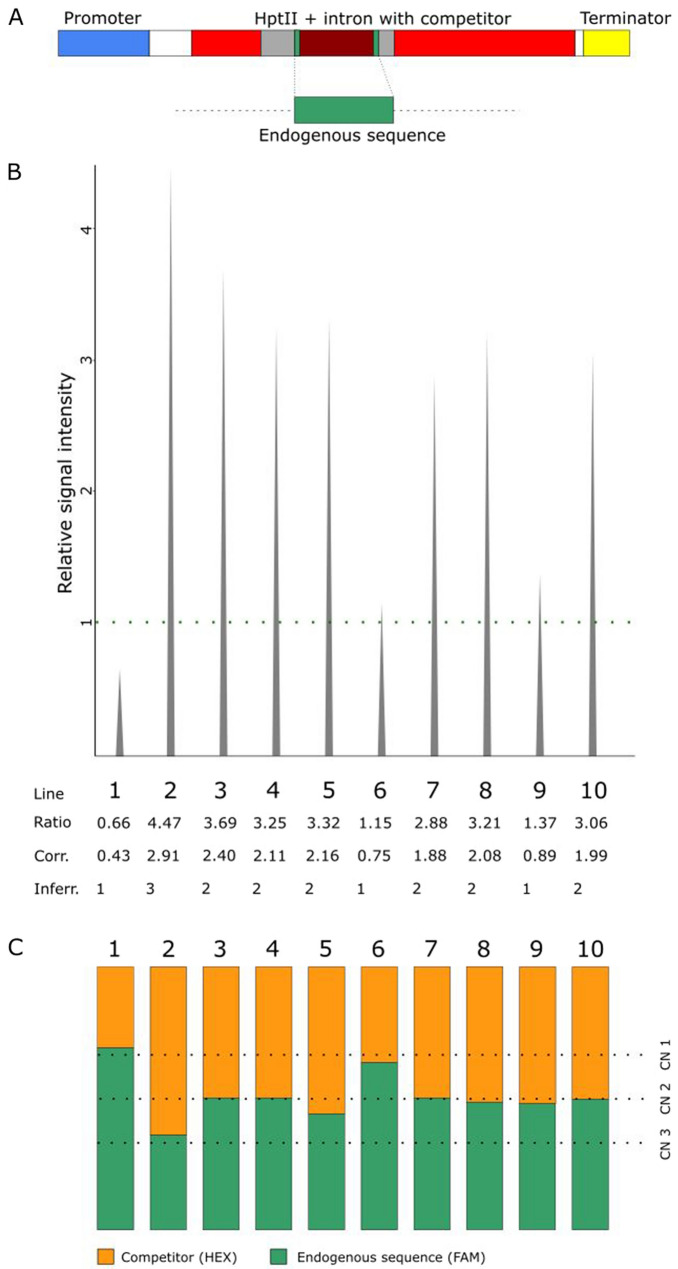
IMPLANT results in T0 of *O. sativa.* **A**. Overview of the modified hygromycin resistance marker in pICSL11059, with indication of the competitor (341 bp) and the amplified fragment of the endogenous *O. sativa* sequence (377 bp). **B**. IMPLANT results using capillary gel electrophoresis. The signal intensity of the competitor is set relative to the intensity of the endogenous sequence (dotted green line). The correction factor was empirically determined and set at 0.65. Corr., corrected; Inferr., inferred. **C**. ddPCR analysis results. Bars show the distribution of the number of molecules of the competitor (orange) and the endogenous sequence (green). The dotted lines indicate the theoretical distribution corresponding to a given copy number

Agriculturally important crops frequently do not make a large number of seeds, which often makes segregation analysis impossible. Therefore, we examined if IMPLANT is also compatible with rice (*Oryza sativa*), one of the world’s most important food crops. We again used pICSL11059 as the backbone vector and this time cloned a complete 341-bp competitor inside of the catalase intron (Fig. 3A; Additional file 1: Figure S2C). This competitor was a randomly chosen sequence on chromosome 1 (GC: 39.9%) that was then flanked with identical primer-binding sites derived from a different 377-bp endogenous sequence that also resides on chromosome 1 and with a similar GC content (40.6%). Because of this difference in size, the endogenous and competitor amplicons can be distinguished after the PCR reaction.

Rice transformants were generated and gDNA was extracted using a commercial silica spin column kit (DNeasy plant mini kit, Qiagen) and used in the PCR reaction. According to the IMPLANT analysis (Fig. 3B) most of the lines contained two T-DNA copies with values ranging between 1.88 and 2.40. Three of the lines (#1, #6, #9) were determined to be single insertion lines, ranging between 0.43 and 0.89, of which line #1 deviated strongly from the expected number of 1. Finally, line #2 was estimated to contain three copies of T-DNA.

To verify the accuracy of our method, we compared the results with those obtained by ddPCR (Fig. 3C). We did not use a restriction enzyme to digest the DNA during the ddPCR run but the values obtained very closely matched the expected ratios. We found that all results were identical except for line #9. With IMPLANT we could detect only one copy, whereas ddPCR detected two. It must be noted however that the probe for the ddPCR analysis lies closer to the T-DNA right border than the PCR amplicon of the IMPLANT reaction (Additional file 1: Figure S2C). Therefore, partial T-DNA copies that are truncated between the ddPCR reverse primer and the IMPLANT forward primer may be detected with ddPCR but not with IMPLANT. To clarify further, we subjected line #9 to Next Generation Sequencing (NGS). The NGS analysis indeed indicated that there is a complex T-DNA integration in this line, including a tandem insertion of the T-DNA with also backbone vector integration (data not shown). Based on the reads, we were however not able to delineate all the junctions with the rice genome. For such complex integrations, it is not unexpected that these methods would give different results, given that different sequences are amplified in each method.

Taken together, these data show that IMPLANT is highly accurate in *O. sativa*. The variation in the obtained IMPLANT inferred values is somewhat higher than those seen in the Arabidopsis data, but single-copy insertions can clearly be distinguished from multiple copy insertions, allowing researchers to select single copy insertion lines for further research.

### IMPLANT is compatible with direct PCR in *O. sativa*

**Fig. 4:**
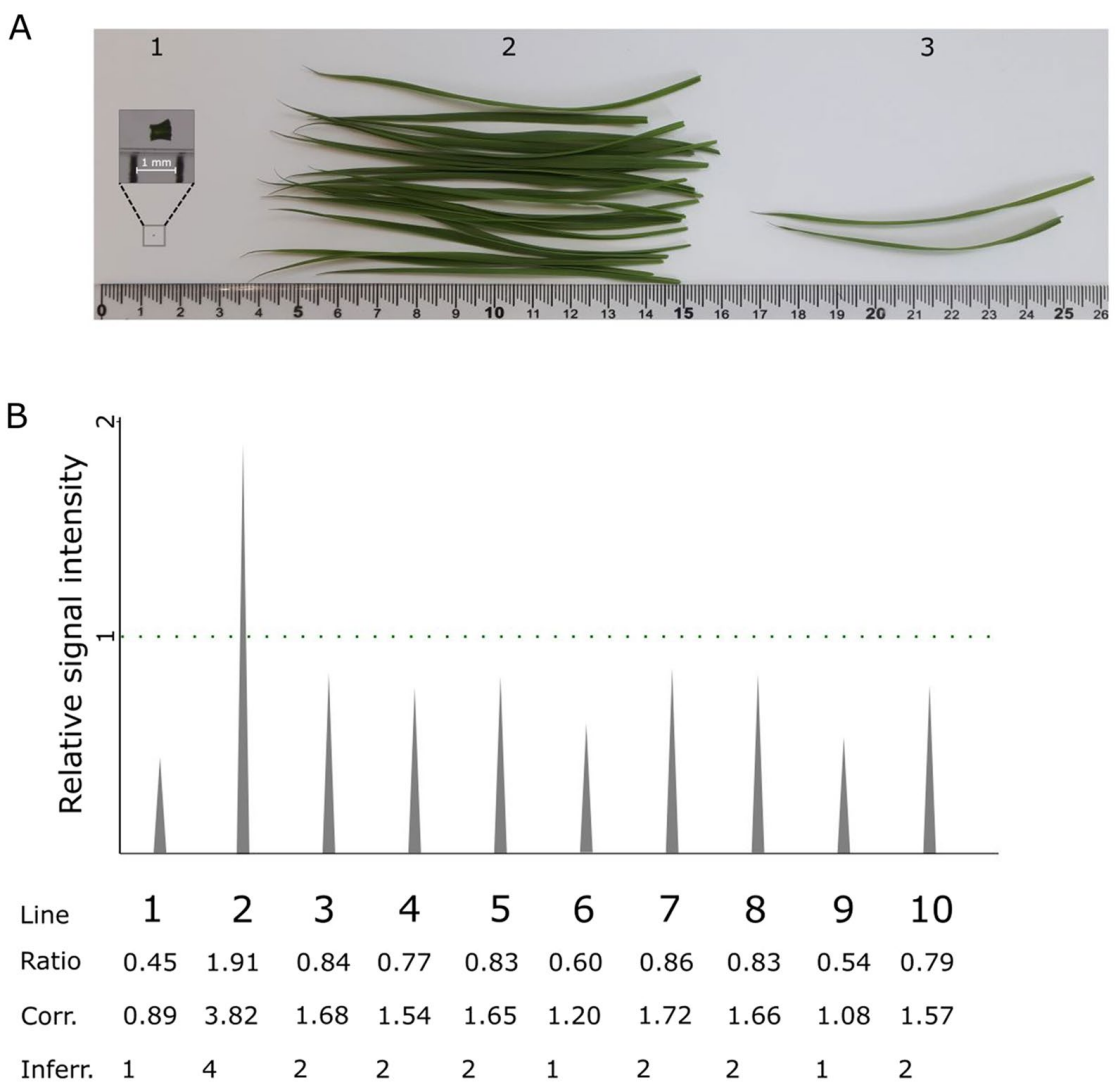
IMPLANT results in *O. sativa* with direct PCR. A. Amount of plant material needed for (1) IMPLANT, which is 0.5 × 0.5 mm^2^ of plant material (magnified in inset), (2) and (3) are upper (500 mg) and lower bounds (33 mg) for generating 10 μg of gDNA with the Qiagen DNeasy Plant Pro Kit. This amount of gDNA suffices for one Southern blot experiment, or for 200 ddPCR reactions. B. IMPLANT direct PCR results. The signal intensity of the competitor is set relative to the intensity of the endogenous sequence (dotted green line). A correction factor of 2 was used for these data. Corr., corrected; Inferr., inferred

To further improve the user-friendliness of our IMPLANT protocol, we investigated if it would be possible to combine it with direct PCR, in which a small amount of plant tissue is directly used as the template without prior DNA extraction. Several methods are available to do so, which use either very small amounts of tissue to avoid the introduction of inhibitors, or by using resistant polymerases [28]. We opted to use the latter system. As can be seen in Fig. 4A, the amount of plant material needed for copy number determination can be drastically reduced by using this direct PCR.

We tested the same 10 plants as in the previous experiment, in which we used silica column-purified DNA, but now we used leaf sections of approximately 0.25 mm^2^ directly as the template in PCR reactions with the Phire Plant Direct PCR kit. As seen in Fig. 4B, this method gives identical results as compared with column-purified DNA (Fig. 3B) for all lines but line #2. The range of values obtained for plants containing one copy was between 0.89 and 1.20, and for plants containing two copies between 1.54 and 1.72, which makes them clearly distinguishable from the single-copy T-DNA lines. The only line that gave a different inferred copy number from the previous experiments is line #2, which gives an apparent copy number of four, whereas the copy number as determined by IMPLANT with purified DNA and ddPCR was three.

We also loaded direct PCR IMPLANT reactions on conventional agarose gel and calculated the band intensities (Additional file 1: Figure S3), giving the same inferred copy numbers as when using the silica column-purified DNA (Fig. 3B). The values for single-copy insertions ranged between 1.02 and 1.12, for plants with two copies, the range was between 1.47 and 2.13, with two of the six lines having a ratio slightly lower than 1.5. Nevertheless, also here, the single-copy lines could be clearly distinguished from these higher-order T-DNA copy lines, making agarose electrophoresis a possible alternative for capillary gel electrophoresis.

In conclusion, direct PCR IMPLANT results corresponded perfectly to the results obtained with the conventional IMPLANT method, except for the line with the highest copy number. The high relative signal intensities of this high copy number line increase the error on the copy number calculation, and therefore make it difficult to accurately distinguish plants containing three or more T-DNAs. However, the most interesting plants, i.e., the individuals with a single copy number, can be perfectly distinguished.

## Discussion

In this paper, we introduced IMPLANT (**I**nsertion of co**m**petitive **P**CR ca**l**ibr**a**tor for copy **n**umber es**t**imation) as a novel technique for copy number estimation. We showed that this technique leads to robust copy number determination in Arabidopsis, and importantly also in rice, an important food crop. Because this method is based on cPCR, only a minute amount of (even low-quality) DNA is needed as opposed to other techniques for copy number determination. Very recently, Fan and coworkers [29] published a conceptually similar method, in which Arabidopsis competitor lines were generated that can be testcrossed with the lines to be tested. The testcrosses are then subjected to cPCR. In our proposed method however, the copy number can be determined directly from the first transformant generation, making it more time efficient, as well as less laborious. As no testcrosses are necessary for IMPLANT, it offers clear advantages for crop plants, for which this is not always straightforward.

We recommend making use of capillary gel electrophoresis to determine the ratio between the competitor and the endogenous sequences after PCR. The main reason for using fragment analysis is that this is typically outsourced, thus avoiding the opening of tubes after amplification in the lab. Indeed, the sensitivity of PCR is a double-edged sword, and contamination of PCR reactions with contaminating amplicons is a real and ever-present danger [30]. This is especially an issue given that this is a quantitative method, and because the same amplicons need to be amplified every time copy numbers are to be determined. In-lab gel electrophoresis is an alternative, albeit one with a risk of carryover contamination. In our preliminary experiments, we also found that gels need to be run for extended periods of time because the size difference between the fragments is only 10%, frequently leading to artifacts. Moreover, when IMPLANT is used with quick-and-dirty DNA preparations or direct PCR, which greatly facilitates the throughput, the likelihood of obtaining a similar amount of amplified DNA at the end of the reaction is low. This in turn leads to differences in gel intensity of the samples, complicating downstream analyses. Next to fragment analysis, other techniques, such as high-resolution melting analysis (HRM) [31] or MALDI-tof (matrix-assisted laser desorption/ionization—time of flight) [32], can also be implemented, depending on the choice of amplicon, risk of contamination, and technical capabilities of the laboratory.

As with any technique, there are a few caveats that need to be considered. First, while powerful, cPCR is a technique with a low dynamic range. This makes it hard to quantify differences that are higher than tenfold [19]. In our experimental setup, the competitor is part of the transgene and subsequently only one concentration of the competitor molecule is used which differs from most other cPCR methods. Consequently, distinguishing differences between three and four insertions seems to be a limit for robust quantification. It must be noted however, that we designed IMPLANT to select lines with a single copy number, which for many applications is ideal. Therefore, the ability to distinguish between one and two copies suffices. For specialized applications, it is also possible to make use of multiple competitors of different lengths (or with other distinguishing characteristics) to improve the dynamic range. Second, because this is a PCR-based technique, the endogenous sequence must be present with intact primer-binding sites. SNPs in, or absence of, primer-binding sites will lead to a lower or abolished amplification. Within the endogenous sequence itself, small point mutations or small indels are tolerable, large deletions or insertions (including T-DNA insertions) are not. This can be mitigated by ensuring the absence of known SNPs and using endogenous sequences that do not tolerate mutations (e.g., haploinsufficient lethal genes). Third, this technique can only detect the presence of a completely integrated competitor; if the DNA is only partially integrated, then it will not be detectable. This drawback is shared with most other techniques for copy number determination. Using two different competitors, at both sides of the integrated fragment, is an option to examine if constructs are fully integrated. Rearrangement of the fragment between the competitors cannot be detected by IMPLANT, however. Fourth, this system is not compatible with twin-T-DNA systems or other systems in which more than one fragment is independently integrated into the genome, unless all fragments contain their own unique competitor. Fifth, like other PCR-based techniques, it cannot distinguish between a double hemizygous T0 plant and a homozygous T1 plant. Hence, the generation of the plants in question needs to be carefully recorded. Sixth, extra sequences need to be introduced into the vector to create the competitor. These sequences can be limited to around 40 bp (the length of 2 primer-binding sites), which should not pose major problems for research purposes or for field release (*cf*. multiple cloning sites that can be present in other vectors). Finally, in our calculations we assumed that the lowest ratios observed in our experiments corresponded to plants containing a transgene copy number of one. This also corresponded with the ddPCR results of the same samples. For researchers wanting to implement IMPLANT, we advise to use a control DNA sample of a known single transgene copy containing plant to determine the correction factor. In this way, the correction factor can be determined free of any assumptions.

While we developed this protocol with plants in mind, this method is applicable to other organisms where the goal is to detect the presence of a low number of insertions. We envision that this protocol will be used not only to routinely determine copy numbers of transgenics, but also for studies that wish to investigate the integration of foreign DNA into the genome. Moreover, this protocol is also perfectly suitable to optimize conditions for obtaining organisms with the required number of DNA insertions. This is relevant for example for the transformation with *Agrobacterium* or other bacteria with the capacity to transfer DNA, where different strains/conditions give rise to different copy numbers [33]. Biolistics, a technique known to frequently give rise to multi-copy transgene-containing plants, can certainly also benefit from this technique [34, 35]. Transgene copy number is also an important consideration for transgenic animals. Extraction of DNA is much more invasive in animals than in plants. The use of blood, ear biopsies, toes, tail tips, the liver (after sacrifice of the animal) and other tissues have been reported [36–38]. The use of IMPLANT, in combination with direct PCR, would be cost effective, fast, and most importantly, improve animal welfare.

In conclusion, our proposed IMPLANT (**I**nsertion of co**m**petitive **P**CR ca**l**ibr**a**tor for copy **n**umber es**t**imation) technique is reliable, and easier, faster and cheaper than other methods for copy determination, and does not require high-quality DNA preparations. Further development of this method by the design of a universal competitor that can be used in a wide variety of plants would greatly facilitate the adoption of this method.

## Materials and methods

### Cloning

For the vector backbone, we made use of pICSL11059, a gift from Nicola Patron (Addgene plasmid # 68263; RRID: Addgene_68263) [27]. This binary vector contains the selectable marker hygromycin phosphotransferase (hpt) with a catalase intron in the coding sequence driven by the Cauliflower mosaic virus 35S promoter and the 5’ untranslated region of the Tobacco Mosaic Virus and terminated by the 35S terminator.

For the cloning of the Arabidopsis competitor, the primers Schlepperless_Pics_Fwd and Schlepperless_Pics_Rev, containing a ClaI and a HindIII restriction site, respectively (Additional file 1: Table S1), were used to amplify a fragment of pICSL11059 while adding the *Schlepperless* primer-binding sequences before the *hpt* start codon and inside the catalase intron sequence (Additional file 1: Figure S1B). The pICSL11059 vector was then linearized in a separate reaction with PICS_Part2_Fwd and PICS_Part2_Rev, and a HindIII restriction site was added during amplification (the ClaI restriction site is already present in the vector). Products of both reactions were then digested with ClaI and HindIII according to standard protocols of New England Biosciences and ligated with T4 ligase. The final construct was Sanger verified with primers PICS_Seq_F and R and transformed into *Agrobacterium tumefaciens* GV3301 using heat shock transformation and selected with rifampicin, gentamicin and carbenicillin.

For the cloning of the *O. sativa* competitor, the primers 301 + 40_Comp_F and 301 + 40_Comp_R were used to add the competitive primer-binding sequences to an *O. sativa* 301-bp fragment to create a 341-bp competitor (Additional file 1: Figure S2A) with a similar GC content as the 377-bp endogenous sequence, which is another random sequence from chromosome 1 of rice (Additional file 1: Figure S2B). The primers 341_Gibson_F and 341_Gibson_R were then used to clone this competitor sequence in the pICSL11059 vector that was linearized using pICS_inverse_F and pICS_inverse_R (Additional file 1: Figure S2C) using TEDA cloning [39]. The final construct was Sanger verified with primers pICS_seq_F and R and transformed into *A. tumefaciens* EHA105 using heat shock transformation and selected with rifampicin and carbenicillin.

### Arabidopsis transformation

Arabidopsis was transformed according to the floral dipping protocol of Logemann and coworkers [40]. Briefly, *Agrobacterium* containing the construct was grown on YEB agar (Rif/Cb) for 2 days at 28 °C. After this, *Agrobacterium* was scraped off the plates and resuspended in 30 mL liquid YEB to an OD_600_ of 2. Per transformation, a 120 mL 5% sucrose solution containing 0.03% of Silwet L-77 was prepared and added into a disposable plastic bag together with 30 mL of the bacteria suspension. The inflorescences of flowering Arabidopsis plants were dipped in this solution for 10 s under gentle agitation. The dipped plants were placed under a lid for 24 h. Plants were then grown until seed set.

### Arabidopsis DNA extraction and PCR

Arabidopsis DNA was extracted according to the protocol of Kasajima and coworkers [25]. Briefly, Edwards solution (200 mM Tris-HCl (pH 7.5), 250 mM NaCl, 25 mM EDTA, and 0.5% SDS) was diluted tenfold in TE buffer (10 mM Tris–HCl (pH 8) and 1 mM EDTA) to obtain extraction buffer. A leaf sample of 1–5 mg was crushed in a 1.5-mL Eppendorf tube together with 200 μL extraction buffer. The solution was centrifuged at 14,000 rpm for 5 min and 1 μL was used as the template in a 25-μL PCR reaction.

For the PCR reactions for the T0 lines, 14.35 μL of H_2_O, 5 μL of GoTaq buffer, 2 μL of dNTPs (2.5 mM each), 1.25 μL of each primer, 1 μL of the template, and 0.15 μL of GoTaq polymerase were added in a PCR tube. For thermocycling the following conditions were used: 94 °C/3 min + 35 x (94 °C/10 s + 55 °C/30 s + 72 °C/60 s) + 72 °C/5 min + 23 °C/∞. Samples were sent to Macrogen (South Korea) for capillary gel electrophoresis; 500LIZ was used as molecular size ladder. Peak Scanner^™^ v1.0 (Thermo Fisher Scientific) was used for determining peak areas. The PCR reactions for the T1 lines were done in a similar fashion, but in this case IP—Taq DNA polymerase (Labopass) was used.

### Arabidopsis segregation analysis

Ripe seeds of the different lines were harvested and gas-sterilized. For gas sterilization, seeds were deposited into Eppendorf tubes and placed in a plastic container together with a beaker with 50 mL of 8% NaOCl. After adding 2 mL of 37% HCl to the beaker, the lid was closed and left for 12 h. Seeds were then placed onto ¼ strength Murashige and Skoog medium (1.15 g MS medium including vitamins (Duchefa), 0.2% Gelrite) containing 30 mg L^−1^ hygromycin B and 100 mg L^−1^ cefotaxime. After 2 d of stratification, they were moved to the growth chamber (21 °C, 16/8 light/dark cycle, 120 μmol/m^2^/s photosynthetic active radiation). After one week of growth, the number of resistant and sensitive seedlings were counted manually. Chi square analysis was done according to Motulsky [41]. Because there are only two phenotypes (resistant and sensitive), one degree of freedom was employed for significance testing. The corresponding value at the 5% significance level is 3.84.

### Rice transformation

Transformation of rice with *A. tumefaciens* was performed according to Toki and coworkers [42] with minor modifications. For the composition of the media, we refer to Toki and coworkers [42]. After sterilizing the seeds, they were inoculated on N6D medium with 0.4% Gelrite for the induction of callus and incubated for 5 days at 32 °C in conditions light conditions (120 µmol/m^2^/s). Bacterial mass of *Agrobacterium* was then scraped from the plate and suspended in AAM medium to yield an OD_600_ of approximately 0.1. The precultured seeds were immersed in this *Agrobacterium* suspension for 1.5 min and afterwards blotted dry on sterile filter paper to remove excess bacteria. The seeds were subsequently transferred onto a sheet of sterilized filter paper (9-cm diameter) that was moisturized with 0.5 mL AAM medium on a plate with 2N6-AS medium. The plates were incubated for 3 days at 25 °C in dark conditions. The seeds were then washed five times with sterilized water and once more in sterilized water with 500 mg L^−1^ carbenicillin to remove the remaining *Agrobacteria*. Afterwards, the seeds were blotted dry and transferred to plates with N6D medium and antibiotics (50 mg L^−1^ hygromycin, 150 mg L^−1^ timentin and 125 mg L^−1^ cefotaxime) for cell selection/proliferation. The plates were incubated for at least 2 weeks at 32 °C in continuous light conditions (120 µmol/m^2^/s). The proliferating calli were then transferred to RE-III media plates supplemented with 50 mg L^−1^ hygromycin for shoot regeneration for 2 weeks or until shoots developed. Plantlets were transplanted into soil and grown until maturity.

### Rice DNA extraction, PCR, and direct PCR

Rice DNA was extracted using the DNeasy plant mini kit (Qiagen) according to the manufacturer’s instructions. For the PCR reaction, 14.35 μL of H_2_O, 5 μL of GoTaq buffer, 2 μL of dNTPs (2.5 mM each), 1.25 μL of each primer, 1 μL of the template (10 ng/μL), and 0.15 μL of GoTaq polymerase were added in a PCR tube. For thermocycling, the following conditions were used: 94 °C/3 min + 35 x (94 °C/10 s + 55 °C/30 s + 72 °C/60 s) + 72 °C/5 min + 23 °C/∞.

For direct PCR, we used the Phire^™^ Plant Direct PCR kit according to the manufacturer’s instructions. For a 20-µL PCR reaction, 10 µL 2 × Phire Plant PCR buffer, 1 µL of both primers, 0.4 µL of Phire DNA polymerase and 7.6 µL of H_2_O were combined. A piece of leaf with the dimensions of 0.50 × 0.50 mm was added to the reaction and the reaction underwent thermocycling with the following conditions: 98 °C/5 min + 40 x (98 °C/5 s + 60 °C/5 s + 72 °C/20 s) + 72 °C/5 min + 23 °C/∞.

Samples were sent to Macrogen (South Korea) for fragment analysis; 500LIZ was used as size standard. Peak Scanner^™^ v1.0 (Thermo Fisher Scientific) was used for determining peak areas. For agarose gel electrophoresis, 5 μL of each sample was mixed with 1 μL of loading dye and separated on a 3% agarose gel until sufficient separation of the bands was obtained. Gels were imaged with a ChemiDoc XRS + system (Bio-Rad) and analyzed using ImageJ.

### IMPLANT data analysis

For samples that were sent for capillary analysis, the peak areas were used to calculate the ratio between the competitor and the endogenous sequence. For samples that were analyzed by conventional agarose electrophoresis, the peak areas were measured by ImageJ using the Analysis tool. To account for differences in amplification efficiency of the competitor and the endogenous gene amplicon, a correction factor was used. The correction factor was adjusted as to maximize the number of individuals in the transgenic population that have expected copy numbers (i.e., as close as possible to integer numbers 1, 2, 3, etc.).

### ddPCR

ddPCR reactions were outsourced to BioD (Seoul, South Korea). Approximately 50 ng of gDNA was used per reaction. Reactions were run on a Clarity^™^ Digital PCR machine using the 2 × qPCRBIO Probe Mix No-ROX (PCR BioSystems). DNA was not digested with restriction enzymes. For the detection, a probe-based system was used. For reference gene amplicons FAM^™^-labeled probes were used, for transgene amplicons HEX^™^-labeled probes. Probes were double-quenched with ZEN^™^ and Iowa Black Hole Quencher^®^ 1. For the primers and probes that were used please refer to Additional file 1: Table S1.

### NGS

One µg of high-quality DNA was sent for NGS sequencing by BGI Genomics (China). The sequencing was done using DNBSeq^™^ paired-end sequencing (150 bp; 10 Gb), and the reads were analyzed by Geneious bioinformatics software.

## Supplementary Information

**Additional file 1: Figure S1.** Arabidopsis IMPLANT sequence information. **Figure S2.** *Oryza* *sativa* IMPLANT sequence information. **Figure S3.** Direct PCR IMPLANT reactions of 10 *O. sativa* lines on conventional 3% agarose gel.A. Agarose gel showing the PCR reactions of the 10 lines, wild type and a negative control. The upper band is the endogenous gene, the lower band is the competitor. B. Gel intensity measurements of the different bands with the calculated peak area, the inferred copy number, and the peak ratio between brackets. An asterisk indicates lines with one T-DNA copy. **Figure S4.** Copy number estimation of T1 Arabidopsis plants using ddPCR and IMPLANT. On the ddPCR graph, the bars show the distribution of the number of molecules of the competitor (orange) and the endogenous sequence (green). The dotted lines indicate the theoretical distribution corresponding to a given copy number. The IMPLANT data are based on capillary gel electrophoresis data. The signal intensity of the competitor is set relative to the intensity of the endogenous sequence. The correction factor was empirically determined and set at 2. Corr., corrected; Inferr., inferred. **Table S1.** Primers and probes used in this study.

## Data Availability

The datasets and vectors used and/or analyses in the underlying study are available from the corresponding author upon reasonable request.
