# Supplementary material for: IMPLANT: a new technique for transgene copy number estimation in plants using a single end-point PCR reaction
Source: Plant Methods. 2022 Dec 9;18:132. doi: 10.1186/s13007-022-00965-0 (PMC9732982; doi:10.1186/s13007-022-00965-0)
Supplement: Supplementary file 1 — Additional file 1: Figure S1. Arabidopsis IMPLANT sequence information. Figure S2. Oryza sativa IMPLANT sequence information. Figure S3. Direct PCR IMPLANT reactions of 10 O. sativa lines on conventional 3% agarose gel.A. Agarose gel showing the PCR reactions of the 10 lines, wild type and a negative control. The upper band is the endogenous gene, the lower band is the competitor. B. Gel intensity measurements of the different bands with the calculated peak area, the inferred copy number, and the peak ratio between brackets. An asterisk indicates lines with one T-DNA copy. Figure S4. Copy number estimation of T1 Arabidopsis plants using ddPCR and IMPLANT. On the ddPCR graph, the bars show the distribution of the number of molecules of the competitor (orange) and the endogenous sequence (green). The dotted lines indicate the theoretical distribution corresponding to a given copy number. The IMPLANT data are based on capillary gel electrophoresis data. The signal intensity of the competitor is set relative to the intensity of the endogenous sequence. The correction factor was empirically determined and set at 2. Corr., corrected; Inferr., inferred. Table S1. Primers and probes used in this study. [file 13007_2022_965_MOESM1_ESM.docx]

**SUPPLEMENTARY MATERIALS**

**Supplementary Figuress**

**A**

TGCAAAGAATCTTCTGGATCCTTTTTCTCTCAGCTACTCTCTCCATTTATCTAATAAACCCTAACCCCTCCTCCCACACTATTCGTTTACACTAACCACTTCCTTATCCACACCAATCCAGAACGTTCCTCAAAAAGCTCCCTCCTTTTCTCTCAAAATGGCGTCTGCAAACGCTCTCTCTTCCGCCTCCGTCCTCTGTTCTTCCCGTCAGGTACAAAGTTTGTTCCTTTTTACCTTCCTTAGCTACTTACTAAAGAAGAAAATGAATCATCTTTCATTACTTTGTTTTGATTGGATTTGACCCTTCAGTGTTATCTATTTTCAAAAACCTTTCTCTATCTGGTTTATTGGGTTTTTGAAGAAAGTGATTGAATTTGACCCTTTAGTTTGTTCTGTTTCAATTCAATTGCTTTTCTCTATTTGGTTTCTGGGTTGGTGAGAAAAGGTTAATTTTTATGGGAATTAGAAGAAACTGATTGAGATTAATGTTAATGAATCTGCTTTATTGATGTTGTGGGTATACAGAGTAAGTTGGGAGGAGGAAATCAGCAGCAAGGTCAGAGAGTTAGCTACAACAAGAGGACAATCAGACGTTTTAGTGTTAGAGCTAATGTAAAGGAAATAGCTTTTGACCAGCACTCTAGAGCTGCTCTACAAGCTGGTATTGATAAGCTTGCTGATTGTGTTGGTCTCACTCTTGGCCCTAGAGGTATTTGTTAATATTAGTAGCTCCTTTACTCTTTCCATCTTCATCTGTGTTATTTATCTTTCGACTTATCTTGGTGTTTTCATTTGTTATTCTTAAGGGAGGAATGTTGTGTTGGATGAATTTGGAAGTCCTAAGGTTGTGAATGATGGAGTCACCATTGCTAGAGCTATTGAGTTACCTAATGCCATGGAAAATGCTGGTGCAGCGCTTATTCGTGAGGTTGGTGTAGCTTTGTTATTGTCTCTGTTAATGCTATTTATACGGTGCGGTAATCTGGTGTTGTTATGTGGTAGGTTGCGAGTAAGACTAATGACTCTGCTGGTGATGGGACAACCACTGCGTCTATCCTTGCTCGGGAGATAATCAAACATGGATTGTTGAGCGTCACTTCTGGTGCGAATCCCGTTTCACTCAAGAGGGGAATTGATAAGACTGTTCAAGGTCTGATCGAAGAGCTTCAGAAGAAAGCTAGGCCCGTGAAAGGTCGTGATGACATCAGAGGTACTAACTTTGCAGTGACCTTCCTTGTGATGCAGCAATGTTTGATTTCTTTTACTGATCAATCTGTTCTTTTTTTTTTTTCCAGCTGTGGCTTCTATCTCTGCTGGAAATGATGACCTTATAGGGTCAATGATTGCTGATGCCATCGACAAAGTTGGACCTGATGGTGTTTTGTCCATTGAATCTTCATCTTCTTTTGAGACCACGGTCGAAGTTGAAGAAGGGATGGAGGTATGAGTTTTTTTGTCTTGCTTCTCTTCAACTGTAATTTTATTCACCATTCATTGCCAATGACTAACTTGATTATTTACAGATTGACAGAGGTTACATCTCGCCTCAGTTTGTTACAAACCCTGAGAAACTACTAGCTGAGTTTGAGAATGCTAGGGTATTGATCACTGATCAGAAGATCACTGCGATCAAAGACATCATCCCTATTTTGGAGAAGACCACTCAGCTTCGTGCTCCGTTGTTGATTATTGCAGAGGATGTTACTGGTGAAGCCTTAGCTACCCTTGTCGTGAACAAGCTTCGTGGTGTCCTCAATGTTGTTGCCGTTAAAGCGCCAGGATTTGGAGAAAGAAGAAAAGCCATGCTTCAAGATATTGCAATCTTGACAGGTATATGCAGATTTCGCGTGCGTTTAGTACTTTTACTTTCTCTCTGTTATGGTCTAAACTTTTTATCATTTTGTATGTTTTTCAGGAGCTGAGTACCTAGCCATGGACATGAGCTTACTGGTTGAAAACGCAACCATAGATCAATTGGGTATTGCCCGAAAAGTCACGATTAGCAAAGATTCGACTACCCTCATTGCAGATGCAGCTTCCAAGGACGAATTACAAGCTCGGATTGCTCAGCTGAAGAAAGAACTATTCGAGACTGATTCTGTGTATGACTCAGAGAAGCTCGCTGAGAGAATTGCGAAGCTATCTGGAGGTGTTGCTGTCATTAAAGTCGGAGCAGCAACTGAAACTGAGCTTGAGGACCGTAAGCTTCGTATCGAGGATGCAAAGAACGCAACATTTGCTGCTATCGAGGAAGGCATAGTTCCAGGTGGTGGTGCTGCTTTGGTGCATCTCTCCACTGTTATTCCCGCCATTAAGGAGACTTTTGAGGATGCTGATGAACGTTTGGGAGCTGACATAGTACAAAAGGTAGCATTTAGTTTTTCGGTTCAGTATTTGTTCTGTAACCAATTGTGACTAGTTATCTTATAAGGGTTTATGTTTTGAGTTCAGGCATTGCTGTCACCAGCTGCTCTTATTGCTCAGAACGCTGGAGTTGAAGGAGAAGTTGTTGTGGAAAAGATTATGTTCAGCGATTGGGAGAATGGGTACAACGCAATGACTGATACATATGAGAATCTGTTCGAAGCTGGAGTGATTGATCCGGCTAAAGTGACAAGATGTGCGCTACAGAACGCTGCTTCCGTAGCAGGAATGGTACTGACCACTCAGGCCATTGTTGTTGACAAACCGAAACCCAAGGCTCCTGCTGCTGCTGCTCCTGAGGGTCTCATGGTGTAATCAAAACACAACATCTTATCATCACAATGGCATCTTTGTCTTTTTGCACATTTGTCATCCCCATTTATCTGCAATGTTTTTCTTTTTTGGGGGAATATGAGTAGTTTGTTAACAGTATCGAGAAATAATTATCATCTTTTTTGTCCAATGGAAATTGCAGAAAATGGTTATGTATGCGACCAAACATTTTATATGTAGCGAGAGTAGTTTAGCAGAACAATAAGCAAATATGCGTACAAACTGAATAATGTTCCAACTTACTATACTATTAAACTAGGAGGGAACAATGTTTTTTCATGCCTTT

TGAGACTTTTCAACAAAGGATAATTTCGGGAAACCTCCTCGGATTCCATTGCCCAGCTATCTGTCACTTCATCGAAAGGACAGTAGAAAAGGAAGGTGGCTCCTACAAATGCCATCATTGCGATAAAGGAAAGGCTATCATTCAAGATCTCTCTGCCGACAGTGGTCCCAAAGATGGACCCCCACCCACGAGGAGCATCGTGGAAAAAGAAGAGGTTCCAACCACGTCTACAAAGCAAGTGGATTGATGTGACATCTCCACTGACGTAAGGGATGACGCACAATCCCACTATCCTTCGCAAGACCCTTCCTCTATATAAGGAAGTTCATTTCATTTGGAGAGGACACGCTCGAGTATAAGAGCTCATTTTTACAACAATTACCAACAACAACAAACAACAAACAACATTACAATTACATTTACAATTATCGATGTCACGATTAGCAAAGATTCGAACAATGAAAAAGCCTGAACTCACGCGACGTCTGTCGAGAAGTTTCTGATCGAAAAGTTCGACAGCGTCTCCGACCTGATGCAGCTCTCGGAGGGCGAAGAATCTCGTGCTTTCAGCTTCGATGTAGGAGGGCGTGGATATGTCCTGCGGGTAAATAGCTGCGCCGATGGTTTCTACAAAGATCGTTATGTTTATCGGCACTTTGCATCGGCCGCGCTCCCGATTCCGGAAGTGCTTGACATTGGGGAATTCAGCGAGAGCCTGACCTATTGCATCTCCCGCCGTGCACAGGGTGTCACGTTGCAAGACCTGCCTGAAACCGAACTGCCCGCTGTTCTGCAGGTAAATTTCTAGTTTTTCTCCTTCGTTTGGGAGCTGACATAGTACATTTTCTTGGTTAGGACCCTTTTCTCTTTTTATTTTTTTGAGCTTTGATCTTTCTTTAAACTGATCTATTTTTTAATTGATTGGTTATGGTGTAAATATTACATAGCTTTAACTGATAATCTGATTACTTTATTTCGTGTGTCTATGATGATGATGATAACTGCAGCCGGTCGCGGAGGCCATGGATGCGATCGCTGCGGCCGATCTTAGCCAGACGAGCGGGTTCGGCCCATTCGGACCGCAAGGAATCGGTCAATACACTACATGGCGTGATTTCATATGCGCGATTGCTGATCCCCATGTGTATCACTGGCAAACTGTGATGGACGACACCGTCAGTGCGTCCGTCGCGCAGGCTCTCGATGAGCTGATGCTTTGGGCCGAGGACTGCCCCGAAGTCCGGCACCTCGTGCACGCGGATTTCGGCTCCAACAATGTCCTGACGGACAATGGCCGCATAACAGCGGTCATTGACTGGAGCGAGGCGATGTTCGGGGATTCCCAATACGAGGTCGCCAACATCTTCTTCTGGAGGCCGTGGTTGGCTTGTATGGAGCAGCAGACGCGCTACTTCGAGCGGAGGCATCCGGAGCTTGCAGGATCGCCGCGGCTCCGGGCGTATATGCTCCGCATTGGTCTTGACCAACTCTATCAGAGCTTGGTTGACGGCAATTTCGATGATGCAGCTTGGGCGCAGGGTCGATGCGACGCAATCGTCCGATCCGGAGCCGGGACTGTCGGGCGTACACAAATCGCCCGCAGAAGCGCGGCCGTCTGGACCGATGGCTGTGTAGAAGTACTCGCCGATAGTGGAAACCGACGCCCCAGCACTCGTCCGAGGGCAAAGGAATAGGCTTCTCTAGCTAGAGTCGATCGACAAGCTCGAGTTTCTCCATAATAATGTGTGAGTAGTTCCCAGATAAGGGAATTAGGGTTCCTATAGGGTTTCGCTCATGTGTTGAGCATATAAGAAACCCTTAGTATGTATTTGTATTTGTAAAATACTTCTATCAATAAAATTTCTAATTCCTAAAACCAAAATCCAGTACTAAAATCCAGATC

**B**

**Figure S1** Arabidopsis IMPLANT sequence information.

A. *SCHLEPPERNESS* (*AT2G28000*) sequence on which the Arabidopsis competitor is based (retrieved from PLAZA Plant 4.0.). Green sequences indicate the UTR, blue the exons and yellow the introns. The primer-binding sites for the amplification of the 370‑bp amplicon are highlighted in red.

B. Hygromycin resistance marker cassette adapted for IMPLANT in Arabidopsis. The turquoise sequence indicates the CaMV 35S promoter, pink the 5’ untranslated region of the Tobacco Mosaic Virus, green the hygromycin phosphotransferase coding sequence, gray the catalase intron and yellow the Cauliflower Mosaic Virus polyA signal. The 408‑bp competitor amplicon is delineated by the primer-binding sites indicated in red. This competitor is approximately 10% larger than the endogenous gene.

cacaattacttcgcttccctgcaacaagcaatttaatcaaataccatagccgataaccaagaccacgccaacggataaaaagatgattaacccaaacataattcgacagtagtacataacatgatttcaaaaccaaaagtagggcatcacaatcttcacagaggtgatgctaaggtgttcagttccaaggagactagtttaatacgacatgacaaaaaaggattaattatcatctaataaccagttcgatttcctcctccttccttacttccgcccccaaagaacaggagcctacgcctaa

**A**

**B**

ttgaaggagggattgcgcttgcaattctcaccgcaatatttcatggtgttgatcccagaacgtctttcctgattggagaaggtttggtgttcagctggtggttgtgtggtacttatacgggcatctttcgccaagaacttcagaggaaataccatctcaaggtgaaatcatgaaaatcctttactgcttcattgtctatattccacttgactgttattcagacaaagtgatcccatgacctgtatattttttatttttggctcaggtagttacactggagacctgtatgaatcattaaacagtattgctttgcaatgttgccttatgctgtgttaaaaatggaatctgaacaaaacctggttgtctcgtggatctgc

**C**

TGAGACTTTTCAACAAAGGATAATTTCGGGAAACCTCCTCGGATTCCATTGCCCAGCTATCTGTCACTTCATCGAAAGGACAGTAGAAAAGGAAGGTGGCTCCTACAAATGCCATCATTGCGATAAAGGAAAGGCTATCATTCAAGATCTCTCTGCCGACAGTGGTCCCAAAGATGGACCCCCACCCACGAGGAGCATCGTGGAAAAAGAAGAGGTTCCAACCACGTCTACAAAGCAAGTGGATTGATGTGACATCTCCACTGACGTAAGGGATGACGCACAATCCCACTATCCTTCGCAAGACCCTTCCTCTATATAAGGAAGTTCATTTCATTTGGAGAGGACACGCTCGAGTATAAGAGCTCATTTTTACAACAATTACCAACAACAACAAACAACAAACAACATTACAATTACATTTACAATTATCGATACAATGAAAAAGCCTGAACTCACCGGACGTCTGTCGAGAAGTTTCTGATCGAAAAGTTCGACAGCGTCTCCGACCTGATGCAGCTCTCGGA**GGGCGAAGAATCTCGTGCTT**TCAGCTTCGATGTAGGAGGGCGTGGATATGTCCTGCGGGTAAATAGCTGCGCCGATGGTTTCTACAAAGATCGTTATGTTTATCGGCACTTTGCATCGGCCGCGCTCCCGATTCCGGAAGTGCTTGACATTGGGGAATTCAGCGAGAGCCTGACCTATTGCATCTCCCGCCGTGCACAGGGTGTCACGTTGCAAGACCTGCCTGAAACCGAACTGCCCGCTGTTCTGCAGGTAAATTTCTAGTTTTTCTCCTTCATTTTCTTGGTTAGGACCCTTTTCTCTTTTTATTTTTTTGAGCTTTGATCTTTCTTTAAACTGATCTATTTTTTAATTGATTGGTTATGGTGTAAATATTACATATTGAAGGAGGGATTGCGCTTcacaattacttcgcttccctgcaacaagcaatttaatcaaataccatagccgataaccaagaccacgccaacggataaaaagatgattaacccaaacataattcgacagtagtacataacatgatttcaaaaccaaaagtagggcatcacaatcttcacagaggtgatgctaaggtgttcagttccaaggagactagtttaatacgacatgacaaaaaaggattaattatcatctaataaccagttcgatttcctcctccttccttacttccgcccccaaagaacaggagcctacgcctaaTGGTTGTCTCGTGGATCTGCGCTTTAACTGATAATCTGATTACTTTATTTCGTGTGTCTATGATGATGATGATAACTGCAGCCGGTCGCGGAGGCCATGGATGCGATCGCTGCGGCCGATCTTAGCCAGACGAGCGGGTTCGGCCCATTCGGACCGCAAGGAATCGGTCAATACACTACATGGCGTGATTTCATATGCGCGATTGCTGATCCCCATGTGTATCACTGGCAAACTGTGATGGACGACACCGTCAGTGCGTCCGTCGCGCAGGCTCTCGATGAGCTGATGCTTTGGGCCGAGGACTGCCCCGAAGTCCGGCACCTCGTGCACGCGGATTTCGGCTCCAACAATGTCCTGACGGACAATGGCCGCATAACAGCGGTCATTGACTGGAGCGAGGCGATGTTCGGGGATTCCCAATACGAGGTCGCCAACATCTTCTTCTGGAGGCCGTGGTTGGCTTGTATGGAGCAGCAGACGCGCTACTTCGAGCGGAGGCATCCGGAGCTTGCAGGATCGCCGCGGCTCCGGGCGTATATGCTCCGCATTGGTCTTGACCAACTCTATCAGAGCTTGGTTGACGGCAATTTCGATGATGCAGCTTGGGCGCAGGGTCGATGCGACGCAATCGTCCGATCCGGAGCCGGGACTGTCGGGCGTACACAAATCGCCCGCAGAAGCGCGGCCGTCTGGACCGATGGCTGTGTAGAAGTACTCGCCGATAGTGGAAACCGACGCCCCAGCACTCGTCCGAGGGCAAAGGAATAGGCTTCTCTAGCTAGAGTCGATCGACAAGCTCGAGTTTCTCCATAATAATGTGTGAGTAGTTCCCAGATAAGGGAATTAGGGTTCCTATAGGGTTTCGCTCATGTGTTGAGCATATAAGAAACCCTTAGTATGTATTTGTATTTGTAAAATACTTCTATCAATAAAATTTCTAATTCCTAAAACCAAAATCCAGTACTAAAATCCAGATC

**Figure S2** *Oryza* *sativa* IMPLANT sequence information.

A. 301‑bp fragment (39.86% GC) from *O. sativa* chromosome 1. This fragment was used to construct the competitor, by flanking this sequence with the primer-binding sites of fragment B.

B. 377‑bp fragment (40.58% GC) from *O. sativa* chromosome 1. This fragment serves as the endogenous sequence. The primer-binding sites are underlined.

C. Hygromycin resistance marker cassette adapted for IMPLANT in *O.* *sativa*. The turquoise sequence indicates the CaMV 35S promoter, pink the 5’ untranslated region of the Tobacco Mosaic Virus, green the hygromycin phosphotransferase II coding sequence, gray the catalase intron and yellow the CaMV polyA signal. The 341‑bp competitor amplicon is delineated by the primer-binding sites indicated in red, the remaining part of the competitor is indicated in dark red. This competitor is approximately 10% smaller than the endogenous gene. Also indicated are the primers for ddPCR (underlined) and the ddPCR probe (bold).


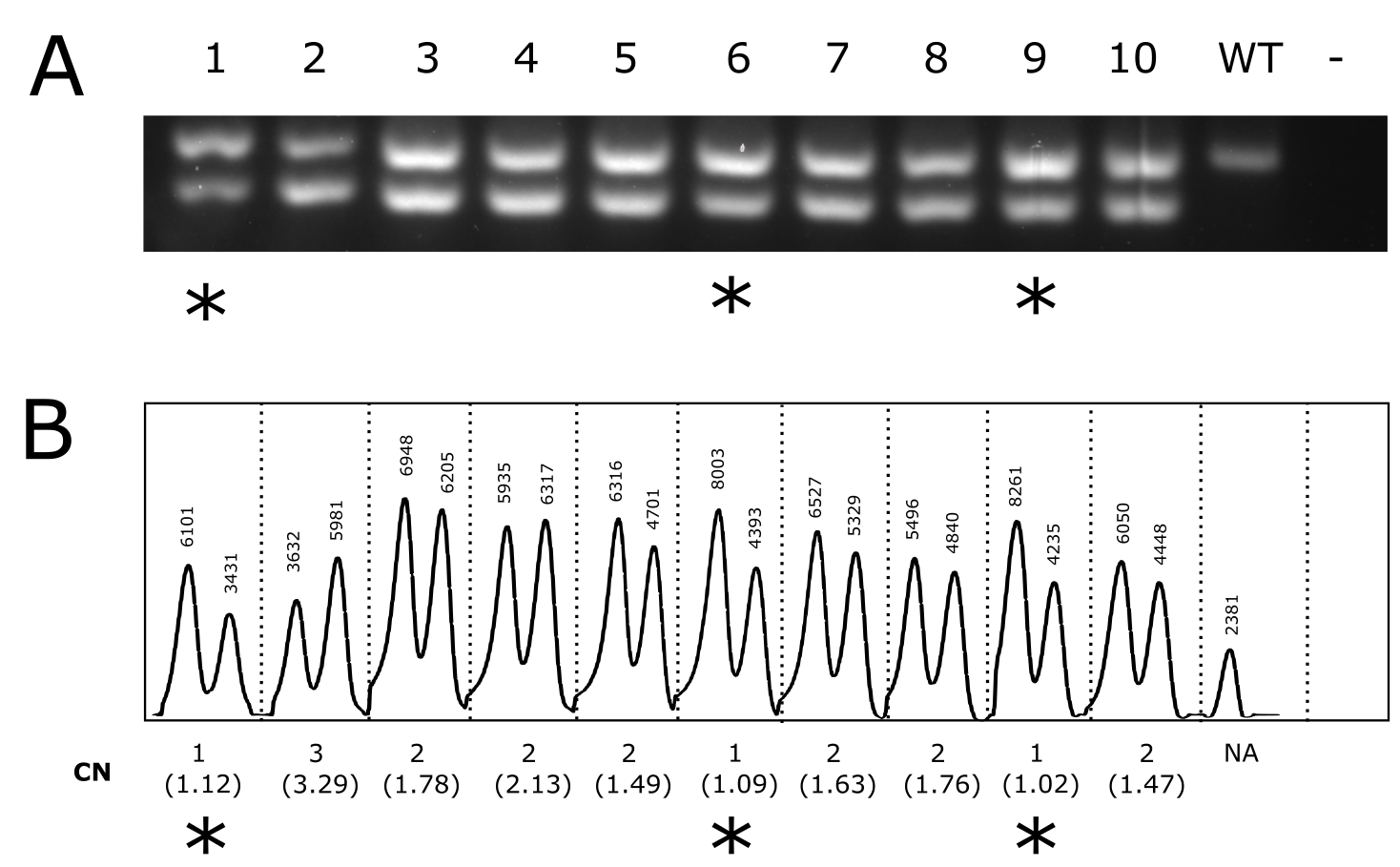


**Figure S3** Direct PCR IMPLANT reactions of 10 *O. sativa* lines on conventional 3% agarose gel. A. Agarose gel showing the PCR reactions of the 10 lines, wild type and a negative control. The upper band is the endogenous gene, the lower band is the competitor. B. Gel intensity measurements of the different bands with the calculated peak area, the inferred copy number, and the peak ratio between brackets. An asterisk indicates lines with one T-DNA copy.

**
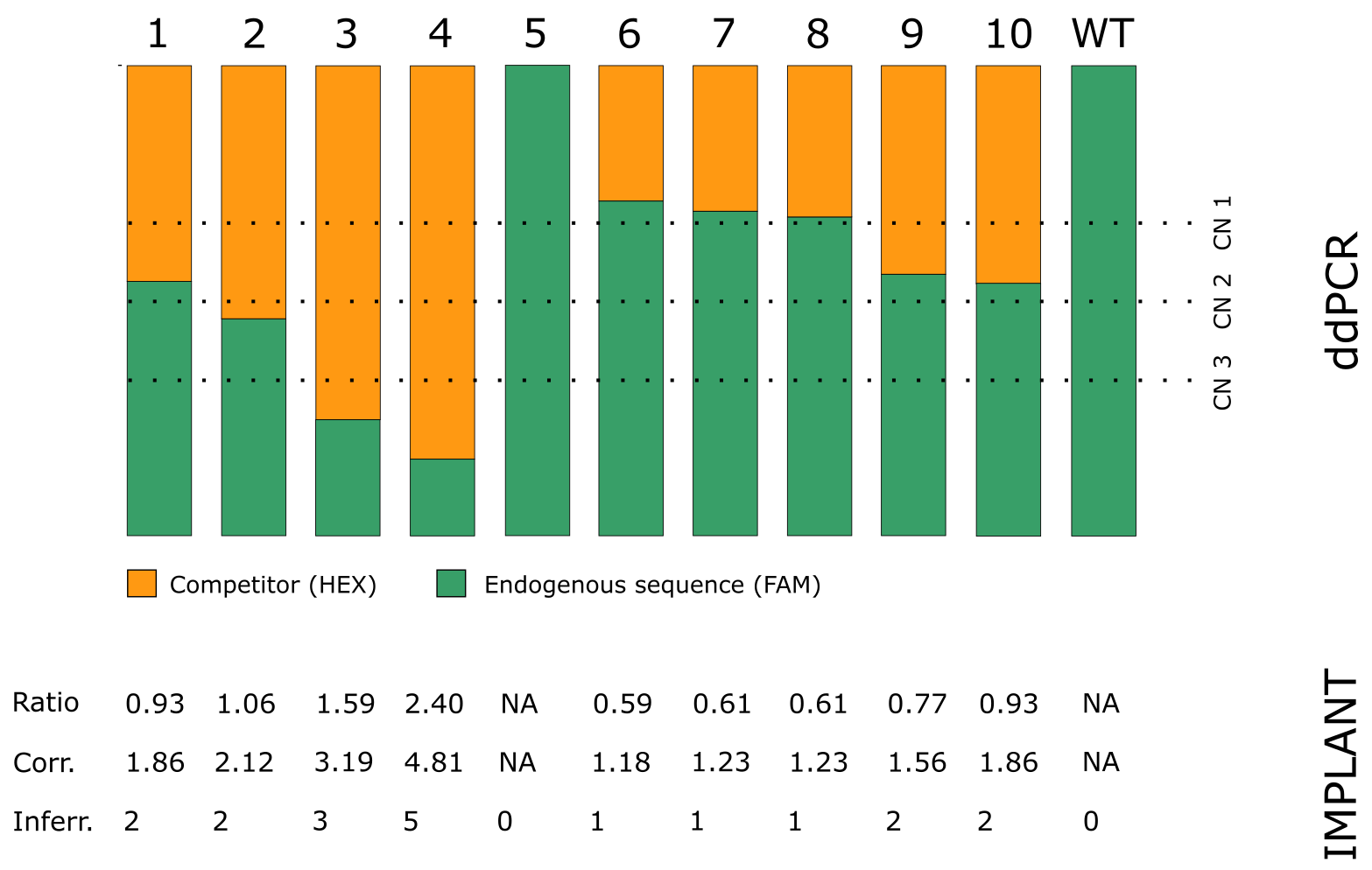
**

**Figure S4** Copy number estimation of T1 Arabidopsis plants using ddPCR and IMPLANT. On the ddPCR graph, the bars show the distribution of the number of molecules of the competitor (orange) and the endogenous sequence (green). The dotted lines indicate the theoretical distribution corresponding to a given copy number. The IMPLANT data are based on capillary gel electrophoresis data. The signal intensity of the competitor is set relative to the intensity of the endogenous sequence. The correction factor was empirically determined and set at 2. Corr., corrected; Inferr., inferred.

**Table S1** Primers and probes used in this study.

| **Primer/probe name** | **Sequence** | **Purpose** |
| --- | --- | --- |
| Schlepperless_Pics_Fwd | AAAAATCGAT**GTCACGATTAGCAAAGATTCGA**ACAATGAAAAAGCCTGAACTC | Amplification of the pICSL11059 fragment with addition of the ClaI site (underlined); the bold sequence is the primer-binding site for Comp_PCR_Ara_Fwd |
| Schlepperless_Pics_Rev | TTTTAAGCTT**GTACTATGTCAGCTCCCAAAC**GAAGGAGAAAAACTAGAAATTTAC | Amplification of the pICSL11059 fragment with addition of the HindIII site (underlined); the bold sequence is the primer-binding site for Comp_PCR_Ara_Rev |
| PICS_Part2_Fwd | GGGAAGCTTATTTTCTTGGTTAGGACCCT | Linearization of pICSL11059 + addition of the HindIII site for Arabidopsis competitor |
| PICS_Part2_Rev | CCCGCTCGTCTGGCTAAGAT | Linearization of pICSL11059 for Arabidopsis competitor |
| Comp_PCR_Ara_Fwd | GTCACGATTAGCAAAGATTCGA | Arabidopsis cPCR |
| Comp_PCR_Ara_Rev | GTACTATGTCAGCTCCCAAAC | Arabidopsis cPCR |
| 301+40_Comp_F | TTGAAGGAGGGATTGCGCTT CACAATTACTTCGCTTCCCTG | Amplification of 341‑bp rice sequence with addition of competitive primer-binding site |
| 301+40_Comp_R | GCAGATCCACGAGACAACCA TTAGGCGTAGGCTCCTGTTCT | Amplification of 341‑bp rice sequence with addition of competitive primer-binding site |
| 341_Gibson_F | TTGATTGGTTATGGTGTAT  TGAGGAGGGATTGCGCTT | TEDA cloning of the 341-bp rice fragment |
| 341_Gibson_R | ATCAGATTATCAGTTAAAGCG  CAGATCCACGAGACAACCA | for TEDA cloning of the 341-bp rice fragment |
| pICS_inverse_F | GCTTTAACTGATAATCTG | Linearization of pICSL11059 for rice competitor |
| pICS_inverse_R | TTTACACCATAACCAATC | Linearization of pICSL11059 for rice competitor |
| pICS_seq_F | CTCGGAGGGCGAAGAATCTC | Sequencing of pICSL11059 |
| pICS_seq_R | CAATGACCGCTGTTATGCGG | Sequencing of pICSL11059 |
| Comp_PCR_Rice_Fwd | TTGAAGGAGGGATTGCGCTT | Rice cPCR |
| Comp_PCR_Rice_Rev | GCAGATCCACGAGACAACCA | Rice cPCR |
| AtHMB1_F | CAGAAAGGTGGGAAAGAGGA | ddPCR Reference gene Arabidopsis |
| AtHMB1_R | TTTGGTTTGTTTGGGTCCTT | ddPCR Reference gene Arabidopsis |
| AtHMB1-Probe† | AGGCACCGGCTGAGAAGCCT | ddPCR Reference gene Arabidopsis |
| OsUBC_F | CCTTCGGAGACACCTTTTGA | ddPCR Reference gene rice* |
| OsUBC_R | TTGAAATGCACATTCGGGTG | ddPCR Reference gene rice* |
| OsUBC-Probe† | CTCCTTCCTCCGCAAGTTCGC | ddPCR Reference gene rice* |
| HptII_F | GAAAAAGCCTGAACTCACCG | ddPCR Transgene* |
| HptII_R | CATATCCACGCCCTCCTAC | ddPCR Transgene* |
| HptII-Probe^‡^ | AAGCACGAGATTCTTCGCCC | ddPCR Transgene* |

^†^ FAM™ and double‐quenched with ZEN™ and Iowa Black Hole Quencher^®^

^‡^ HEX™ and double‐quenched with ZEN™ and Iowa Black Hole Quencher^®^

*Sequences from Collier et al. (2017)
